# Sperm DNA fragmentation index affect pregnancy outcomes and offspring safety in assisted reproductive technology

**DOI:** 10.1038/s41598-023-45091-6

**Published:** 2024-01-03

**Authors:** Fei Li, Xiaoyan Duan, Mingming Li, Xing Ma

**Affiliations:** 1 Department of Gynaecology and Obstetrics, The First People’s Hospital of Shangqiu, Henan, People’s Republic of China; 2 Graduate School of Zhengzhou University, Henan, People’s Republic of China

**Keywords:** Infertility, Urogenital reproductive disorders

## Abstract

The role of sperm DNA fragmentation index (DFI) in investigating fertility, embryonic development, and pregnancy is of academic interest. However, there is ongoing controversy regarding the impact of DFI on pregnancy outcomes and the safety of offspring in the context of Assisted Reproductive Technology (ART). In this study, we conducted an analysis of clinical data obtained from 6330 patients who underwent in vitro fertilization (IVF) or intracytoplasmic sperm injection (ICSI) at the reproductive medical center of The First People's Hospital of Shangqiu and The Affiliated Hospital of Zhengzhou University. The patients was stratified into two distinct groups: IVF group and ICSI group, Within each group, patients were further classified into three subgroups. IVF: group A (< 15%) included 3123 patients, group B (15–30%) included 561 patients, and group C (≥ 30%) included 46 patients. ICSI: group A (< 15%) included 1967 patients, group B (15–30%) included 462 patients, and group C (≥ 30%) included 171 patients. Data were collected and subjected to statistical analysis. There were no significant differences in the basic characteristics among the three groups, and the sperm DFI did not significantly affect the fertilization rates, pregnancy rates, stillbirth rates and the number of birth defects. However, the incidences of miscarriage rates in IVF/ICSI groups with DFI > 30% and DFI 15–30% were significantly higher than those in IVF/ICSI groups with DFI < 15%, and the miscarriage rates in ICSI group with DFI > 30% were significantly higher than DFI 15–30% group, the smooth fitting curve shows that there is a positive correlation between miscarriage rates and sperm DFI (OR 1.095; 95% CI 1.068–1.123; P < 0.001). The birth weight of infants in the IVF/ICSI groups with DFI > 30% and DFI 15–30% exhibited a statistically significant decrease compared to those in the IVF/ICSI groups with DFI < 15%. Furthermore, the birth weight of infants in the ICSI group with DFI > 30% was lower than that of the DFI 15–30% group. The smooth fitting curve analysis demonstrates a negative association between birth weight and sperm DFI (OR 0.913; 95% CI 0.890–0.937; P < 0.001). Sperm DFI has an impact on both miscarriage rates and birth weight in assisted reproductive technology. The smooth fitting curve analysis reveals a positive correlation between miscarriage rates and DFI, while a negative correlation is observed between birth weight and DFI.

## Introduction

The sperm DNA fragmentation index (DFI) serves as a metric for evaluating the integrity of sperm DNA by quantifying the level of chromatin fragmentation within sperm cells. This index has emerged as a novel marker for assessing sperm quality, with particular relevance to embryonic development and pregnancy outcomes^1,2^. Previous studies have shown that the chromosomes in sperm carry the body's genetic information, and a normal sperm's DNA double strand should be intact and free of debris^3^. Currently, it is believed that a DFI ≤ 15% is considered normal, while 15% < DFI < 30% is considered average. If the DFI ≥ 30%, it is considered to have poor integrity, although a high sperm fragmentation rate may influence pregnancy outcomes and offspring safety, the nature of this relationship remains controversial^4^. The actual impact of sperm DFI on assisted reproductive technology outcomes remains to be fully understood.

Generally, sperm DFI is commonly assessed in studies related to embryonic development, fertilization, pregnancy rates, aneuploidy, and abortions^5–7^, however, there are relatively few studies that have examined the effects of sperm DFI on offspring safety. Dai et al. found a positive correlation between sperm DFI and spontaneous abortion^8^, Conversely, Antonouli et al. reported that sperm DFI did not have a significant impact on embryonic development or pregnancy rate in patients undergoing intracytoplasmic sperm injection (ICSI) with donated oocytes^9^. Additionally, Chen et al. conducted a study demonstrating that sperm DFI did not significantly affect various reproductive outcomes, including fertilization, clinical pregnancy, miscarriage, ongoing pregnancy, or birthweight, across different groups^10^. However, the findings continue to be a subject of debate as many of these studies have lacked standardized measures or had insufficient sample sizes to accurately assess the impact on pregnancy outcomes and the safety of offspring. Consequently, a rigorous investigation with a substantial sample size and extended study duration is imperative to establish a definitive correlation.

In our reproductive medical center, more than ten thousand in vitro fertilization/intracytoplasmic sperm injection (IVF/ICSI) cycles annually, we possess the necessary resources to test the proposed hypothesis. Furthermore, a smooth fitting curve will be developed to evaluate the potential influence of DFI on pregnancy outcomes and offspring safety. To the best of our knowledge, this study represents the first attempt to establish such a curve and explore the relationship between DFI and these outcomes. The findings of this study have the potential to provide pregnant couples with more precise and comprehensive information regarding pregnancy outcomes and offspring safety through the assessment of DFI.

## Materials and methods

### Ethical approval and informed consent

This study was approved by the Ethics Committee of the First People's Hospital of Shanqiu. Due to the retrospective nature of the study (the Ethics Committee of Reproductive Center, the First People's Hospital of Shangqiu) waived the need of obtaining informed consent. All methods were performed in accordance with the relevant guidelines and regulations.

### Subjects

The eligible subjects consisted of 6330 patients who undergoing IVF/ICSI in reproductive medical center of The First People's Hospital of Shangqiu and The Affiliated Hospital of Zhengzhou University between June 2013 to November 2020. The eligible subjects were categorized into two groups, IVF and ICSI, based on different ART methods. The IVF group consisted of 3730 cases, representing 58.93% of the total sample, while the ICSI group comprised 2660 cases, accounting for 41.07%. Subsequently, the patients were further divided into three groups based on the DNA fragmentation index (DFI) value of sperm. In the IVF group, group A (< 15%) included 3123 cases, accounting for 49.34% of the total sample, group B (15–30%) consisted of 561 cases, representing 8.86%, and group C (≥ 30%) comprised 46 cases, accounting for 0.73%. In the ICSI group, group A (< 15%) included 1967 cases, representing 31.07%, group B (15–30%) consisted of 462 cases, accounting for 7.30%, and group C (≥ 30%) comprised 171 cases, accounting for 2.70%. The data were collected from the Clinical Medicine Management System (CCRM), for patient privacy reasons, personal information about participants was not included in the data. The baseline characteristics of patients were female age, male age, duration of infertility, female body mass index (BMI), male BMI, thickness of endometrium, basal FSH, basal LH, basal E2, basal P, AMH and AFC.

We conducted a comparative analysis of pregnancy outcomes and offspring safety data among patients undergoing IVF/ICSI, categorized into three groups based on sperm DNA fragmentation index (DFI): the “DFI < 15% group,” the “DFI 15–30% group,” and the “DFI ≥ 30% group”. Additionally, we identified statistically significant factors within these groups. The pregnancy outcomes of patients were oocyte number, mature oocytes, oocyte maturation rates, transferable embryos, ET cancellation, fertilization rates, pregnancy rates per transfer, miscarriage rates. The offspring safety of patients were rates of stillbirths, prematurity rates, birth weight, LBW rates, birth defect rates. In addition, we further establish a smooth fitting curve to assess whether DFI can affect pregnancy outcomes and offspring safety.

**Figure 1:**
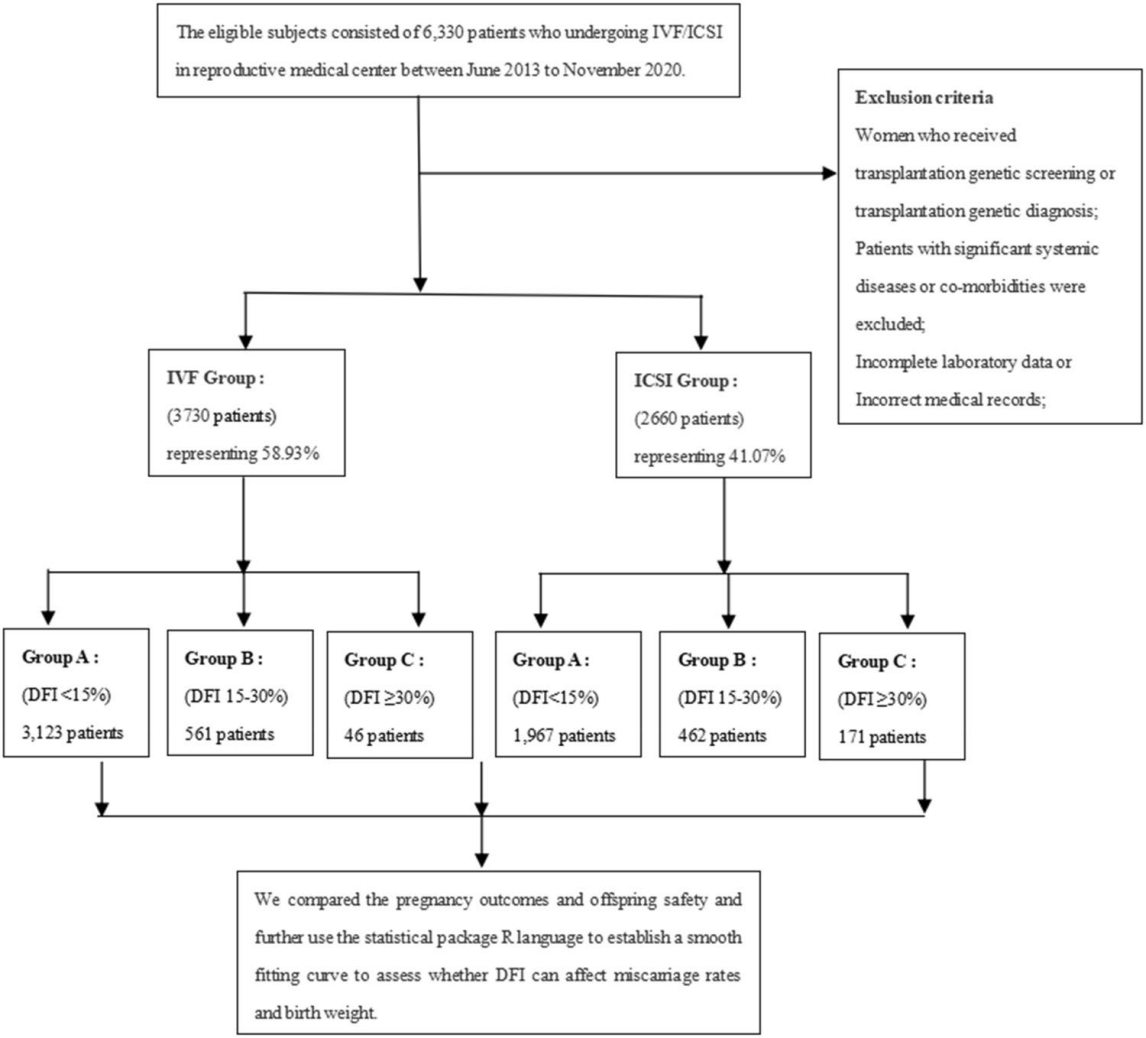
The map of technical route.

All patients undergoing assisted reproductive technology were administered either the early-follicular-phase long-acting GnRH-agonist long protocol or the GnRH antagonist protocol. The clinical pregnancy rate was determined by dividing the number of patients with detected gestational sac/s after embryo transfer by the total number of patients who underwent embryo transfer. Clinical miscarriage rates were calculated by dividing the total number of patients with detectable gestational sacs by the total number of miscarriages before 20 weeks of gestation. Low birth weight infants are defined as birth weights below 2500 g. The map of technical route was shown in Fig. 1.

### Statistical analyses

All analyses were performed using SPSS 26.0 software (IBM, Armonk, NY, USA) and the statistical package R (The R Foundation; http://www.r-project.org; version 3.6.1). Continuous variables are presented as the mean ± SD, and categorical variables are presented as frequencies (percentages). Comparisons among different groups were performed with one-way ANOVA, Kruskal–Wallis test, Chi-square test and Bonferroni-adjusted test. Data analyzed for statistical significance were subjected to a statistical package R3.6.1 software analysis, we use smoothing function curve to further examine the actual relationship between sperm DFI and miscarriage rates and birth weight in assisted reproductive technology.

### Institutional review board statement

Due to the retrospective nature of the study (the Ethics Committee of Reproductive Center, the First People's Hospital of Shangqiu) waived the need of obtaining informed consent.

## Results

We collected eligible data from 6330 patients, The sperm DFI from patients undergoing IVF/ICSI were divided into three groups according to a cutoff established, IVF: group A (< 15%) consisting of 3123 patients, group B (15–30%) of 561 patients and group C (≥ 30%) of 46 patients. ICSI: group A (< 15%) consisting of 1967 patients, group B (15–30%) of 462 patients and group C (≥ 30%) of 171 patients. There were no significant differences in the basic characteristics (female age, male age, duration of infertility, female BMI, male BMI, thickness of endometrium, basal FSH, basal LH, basal E2, basal P, AMH and AFC) among the three groups of IVF/ICIS (Table 1).

**Table 1:** Characteristics of IVF/ICSI patients according to sperm DFI.

Group	IVF patients				ICSI patients			
	DFI < 15% (n = 3123)	DFI15-30% (n = 561)	DFI ≥ 30% (n = 46)	*P* value	DFI < 15% (n = 1967)	DFI15-30% (n = 462)	DFI ≥ 30% (n = 171)	*P* value
Female age (years)	29.63 ± 3.64	30.51 ± 3.93	30.82 ± 3.95	0.083	29.64 ± 4.31	30.22 ± 4.64	30.44 ± 5.10	0.118
Male age (years)	30.06 ± 2.74	30.32 ± 2.94	31.72 ± 2.73	0.142	31.13 ± 3.64	31.38 ± 3.09	31.66 ± 3.72	0.293
Duration of infertility (years)	3.15 ± 1.14	3.53 ± 1.35	3.87 ± 1.28	0.187	3.65 ± 1.32	3.74 ± 1.46	3.88 ± 1.64	0.764
Female BMI (kg/m2)	23.52 ± 3.33	23.62 ± 3.55	23.15 ± 3.01	0.772	24.23 ± 2.92	20.29 ± 3.48^a^	24.82 ± 2.93^b^	0.029
Male BMI (kg/m2)	23.30 ± 2.97	24.08 ± 2.75	24.08 ± 3.53	1.342	24.31 ± 3.09	25.14 ± 2.98	25.16 ± 2.59	1.457
Thickness of endometrium (mm)	9.82 ± 1.91	10.04 ± 1.98	9.29 ± 2.71	0.353	9.43 ± 2.38	9.45 ± 2.43	9.56 ± 3.21	0.893
Basal FSH (IU/L)	6.52 ± 1.65	5.11 ± 2.52^a^	6.92 ± 1.99^b^	0.012	6.91 ± 2.22	7.12 ± 2.45	7.31 ± 2.41	0.712
Basal LH (IU/L)	5.38 ± 3.17	6.66 ± 3.16	5.17 ± 2.37	2.242	5.62 ± 2.94	5.51 ± 2.44	5.91 ± 2.49	2.045
Basal E2 (ng/L)	44.81 ± 31.2	40.25 ± 23.2	45.83 ± 38.4	1.794	41.41 ± 35.33	42.45 ± 29.0	41.58 ± 29.1	1.914
Basal P (μg/L)	0.59 ± 0.52	0.56 ± 0.71	0.54 ± 0.33	0.413	0.51 ± 0.43	0.48 ± 0.46	0.57 ± 0.31	0.311
AMH (ng/mL)	3.41 ± 2.21	3.33 ± 2.67	3.75 ± 3.31	0.422	2.77 ± 1.55	2.82 ± 1.65	2.74 ± 2.42	0.527
AFC (numbers)	15.19 ± 6.55	15.35 ± 6.17	14.97 ± 7.58	0.958	14.18 ± 5.62	14.83 ± 6.26	14.21 ± 6.01	1.287

Data are shown as means ± standard deviation. *BMI* body mass index, *FSH* follicular-stimulating hormone, *LH* luteinizing hormone, *E2* estradiol, *P* progesterone, *AMH* anti-Müllerian hormone, *AFC* antral follicle counting; ^a^*P* < 0.05, vs. DFI < 15%; ^b^*P* < 0.05, vs. DFI 15–30%.

There were no significant differences in the number of oocyte, mature oocytes, maturation rates, transferable embryos, ET cancellation, fertilization rates and pregnancy rates among the three groups of IVF/ICIS. However, the incidences of miscarriage rates in IVF/ICSI groups with DFI > 30% and DFI 15–30% were significantly higher than those in IVF/ICSI groups with DFI < 15% (*P* = 0.005), and the miscarriage rates in ICSI group with DFI > 30% were significantly higher than DFI 15–30% group (*P* < 0.001) (Table 2).

**Table 2:** Pregnancy outcomes of IVF/ICSI patients according to sperm DFI.

Group	IVF patients				ICSI patients			
	DFI < 15% (n = 3123)	DFI15-30% (n = 561)	DFI ≥ 30% (n = 46)	*P* value	DFI < 15% (n = 1967)	DFI15-30% (n = 462)	DFI ≥ 30% (n = 171)	*P* value
Oocyte number	13.03 ± 7.42	12.64 ± 6.75	13.06 ± 7.11	1.432	14.52 ± 6.89	13.23 ± 7.46	13.62 ± 7.02	0.921
No. of mature oocytes	11.03 ± 5.49	11.16 ± 5.14	11.30 ± 5.72	2.088	11.93 ± 6.04	10.42 ± 5.41	11.34 ± 5.21	0.571
Oocyte maturation rates (%)	84.51 ± 23.82	88.37 ± 28.22	86.54 ± 34.82	3.972	82.23 ± 25.15	78.82 ± 32.2	83.3 ± 31.94	1.732
Transferable embryos	9.21 ± 1.64	9.61 ± 1.69	8.72 ± 1.12	1.531	9.32 ± 1.44	9.42 ± 1.92	9.11 ± 1.14	2.125
ET cancellation (%)	16.71 (522/3123)	17.29 (97/561)	17.39 (8/46)	0.939	17.03 (335/1967)	16.45 (76/462)	18.71 (32/171)	0.621
Fertilization rates (%)	63.47 ± 27.51	56.84 ± 30.51	61.05 ± 39.95	0.265	60.34 ± 32.27	55.63 ± 37.45	60.59 ± 38.32	0.192
Pregnancy rates per transfer (%)	47.97 (1498/3123)	46.88 (263/561)	43.48 (20/46)	0.754	48.91 (962/1967)	47.19 (218/462)	43.86 (75/171)	0.359
Miscarriage rates (%)	13.75 (206/1498)	20.15 (53/263)^a^	30.00 (6/20)^a^	0.005	13.62 (131/962)	19.72 (43/218)^a^	33.34 (25/75)^a,b^	< 0.001

Data are shown as means ± standard deviation or frequencies (percentages). ET cancellation, embryo transplantation cancellation. ^a^*P* < 0.05, vs. DFI < 15%; ^b^*P* < 0.05, vs. DFI 15–30%.

Likewise, the sperm DFI were divided into three groups according to IVF and ICSI, IVF: group A (< 15%) consisting of 1292 patients, group B (15–30%) of 210 patients and group C (≥ 30%) of 14 patients. ICSI: group A (< 15%) consisting of 831 patients, group B (15–30%) of 175 patients and group C (≥ 30%) of 50 patients. The sperm DFI did not significantly affect the stillbirths rates, prematurity rates and birth defect rates. However, the birth weight in IVF/ICSI groups with DFI > 30% and DFI 15–30% were significantly lower than those in IVF/ICSI groups with DFI < 15%, and the birth weight in ICSI group with DFI > 30% were lower than DFI 15–30% group (Table 3).

**Table 3:** Offspring safety of IVF/ICSI patients according to sperm DFI.

Group	IVF patients				ICSI patients			
	DFI < 15% (n = 1292)	DFI15-30% (n = 210)	DFI ≥ 30% (n = 14)	*P* value	DFI < 15% (n = 831)	DFI15-30% (n = 175)	DFI ≥ 30% (n = 50)	*P* value
Stillbirths rates (%)	0.31 (4/1292)	0.48 (1/210)	0.00 (0/14)	0.551	0.48 (4/831)	0.57 (1/175)	2.00 (1/50)	0.305
Prematurity rates (%) (< 37 weeks)	20.42 (263/1288)	16.27 (34/209)	21.43 (3/14)	0.372	19.11 (158/827)	17.82 (31/174)	22.45 (11/49)	0.763
Birth weight (grams)	2824.9 ± 723.64	2631.2 ± 701.39^a^	2592.7 ± 678.52^a^	< 0.001	2852.3 ± 741.41	2637.2 ± 711.28^a^	2523.2 ± 692.77^a,b^	< 0.001
LBW rates (%) (< 2500 g)	21.58 (278/1288)	22.01 (46/209)	21.43 (3/14)	0.982	21.40 (177/827)	24.71 (43/174)	38.78 (13/49)	0.479
birth defect rates (%)	0.54 (7/1288)	0.48 (1/209)	7.14 (1/14)	0.105	0.60 (5/827)	1.15 (2/174)	2.04 (1/49)	0.168

Data are shown as means ± standard deviation or frequencies (percentages). *LBW* low birth weight; Birth defects: Down Syndrome; Abnormality of the external auditory canal; Cleft soft palate; Congenital heart disease; Joint deformity of the index and middle fingers; ^a^*P* < 0.05, vs. DFI < 15%; ^b^*P* < 0.05, vs. DFI 15–30%.

**Figure 2:**
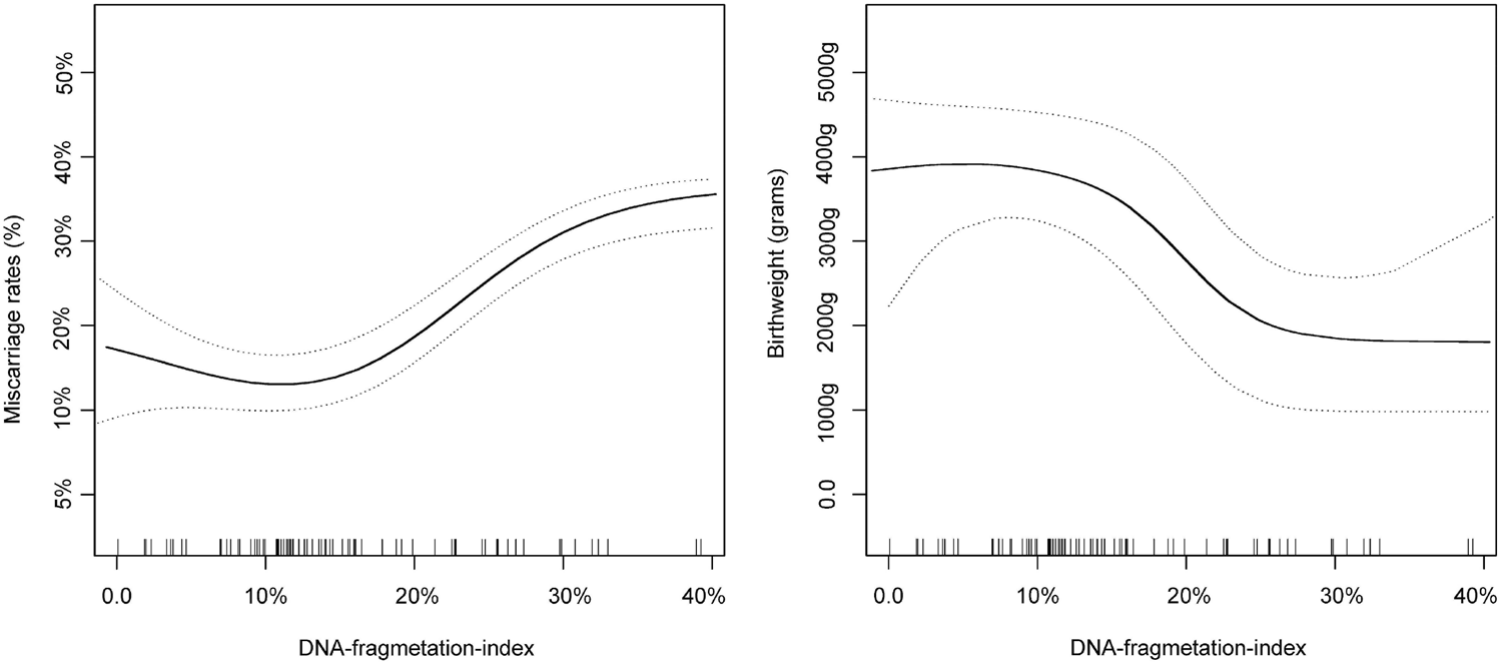
Left graph: association between sperm DFI and miscarriage rates. A nonlinear association between sperm DFI and miscarriage rates was found in a generalized additive model. Right graph: association between sperm DFI and birth weight. Solid line represents the smooth curve fit between variables. Dotted line represent the 95% of confidence interval from the fit.

We further use the statistical package R language to establish a smooth fitting curve to assess whether DFI can affect miscarriage rates and birth weight, the smooth fitting curve shows that there is a positive correlation between miscarriage rates and sperm DFI (OR 1.095; 95% CI 1.068–1.123; *P* < 0.001), the smooth fitting curve shows that there is a negative correlation between birth weight and sperm DFI (OR 0.913; 95% CI 0.890–0.937; *P* < 0.001) (Fig. 2).

## Discussion

Our research findings provide evidence that sperm DNA fragmentation index (DFI) has an impact on both miscarriage rates and birth weight in the context of assisted reproductive technology. Furthermore, the smooth fitting curve analysis demonstrates a positive correlation between miscarriage rates and sperm DFI, as well as a negative correlation between birth weight and sperm DFI. This outcome is of particular interest. To the best of our knowledge, this study may be the first to establish a smooth fitting curve to evaluate the relationship between these variables. The findings of this study can provide valuable insights for clinicians in enhancing their understanding of pregnancy outcomes and ensuring the safety of offspring based on sperm DNA fragmentation index. Previous research has indicated that spermatozoa with elevated DNA damage (DFI) may contribute to reduced rates of fertilization, cleavage, and embryo development^11,12^. Zheng et al. have reported a negative association between high DFI in sperm and the success of in vitro fertilization and embryo transfer (IVF-ET)^13^. Additionally, Dai et al. have found a positive correlation between sperm DFI and the occurrence of spontaneous abortion^8^. These findings may contribute to a better comprehension of the association between DFI and embryo and pregnancy outcomes. However, it is important to note that these studies suffer from limitations such as the absence of standardized measures or insufficient sample sizes, thus potentially compromising the accuracy of the findings in reflecting the relationship between DFI and pregnancy outcomes. In our study, we addressed these limitations by employing a larger sample size and a longer study period. Furthermore, our research expands the existing knowledge on the safety of offspring in relation to DFI, thereby enhancing the credibility of our conclusions. Consequently, pregnant couples can benefit from more precise and comprehensive information regarding pregnancy outcomes and the safety of their offspring.

The measurement of sperm DFI serves as an indicator of sperm DNA integrity and damage, making it a valuable parameter in the investigation of fertility^14,15^. It is widely recognized that the majority of sperm possess a DFI ranging from 0.07 to 0.15, and DFI has emerged as a novel marker for assessing sperm quality and fertility^16,17^. Oxidative stress is a prominent contributor to elevated DFI, thereby diminishing the fertilization capacity of sperm^18^. Varicocele, a condition marked by dilated and convoluted veins in the scrotum, represents a primary risk factor for oxidative stress and subsequent high DFI. Varicocele can cause a buildup of heat and toxins within the scrotum, leading to poor semen quality and oxidative stress^19^. Extensive evidence suggests that varicocelectomy, a surgical intervention aimed at rectifying the varicocele, effectively restores semen parameters to normal levels and significantly reduces DFI in the majority of male patients^18,20^. By eliminating the source of oxidative stress, varicocelectomy holds the potential to enhance fertility outcomes among individuals with elevated DFI. In addition, various reproductive toxic agents can exhibit cell-specific effects on ejaculated sperm, leading to increased DNA fragmentation^21^, consequently, the occurrence of DNA strand breaks in mature sperm is likely attributable to a diverse range of mechanisms^22,23^. Nevertheless, understanding the precise mechanism underlying sperm DNA fragmentation may not be imperative for making clinical decisions. Rather, the focus should be on formulating a comprehensive approach to ensure the well-being of pregnant women exhibiting elevated levels of sperm fragmentation and their offspring. By assessing the proportion of sperm with compromised DNA in an ejaculate, clinicians can provide more informed guidance and develop tailored treatment strategies.

The escalating prevalence of infertility, impacting a substantial population of 80–150 million individuals worldwide, has emerged as a pressing concern in recent decades^24,25^. Assisted Reproductive Technology (ART) is commonly employed as a therapeutic approach for addressing human infertility. While ART offers numerous benefits, it is not without drawbacks, including the occurrence of implantation failures and miscarriages in a significant proportion of embryos^26,27^. In recent times, there has been a growing focus on paternal factors and their influence on embryo quality, particularly in relation to abnormal gametes from parents. Previous research has investigated the correlation between seminal quality, encompassing sperm concentration, motility, and morphology, and the outcomes of ART. It has been observed that inadequate seminal quality, characterized by low sperm concentration and motility, may lead to failures in embryo development^28–30^. In the context of IVF/ICSI cycles, the DFI exhibits a negative correlation with both embryo development and implantation rates, serving as an indicator of sperm chromatin integrity^31,32^. Our study provides further evidence that sperm DFI significantly impacts miscarriage rates and birth weight in assisted reproductive technology, aligning with the findings of numerous prior studies. Furthermore, it is worth noting that the mitochondrial DNA copy number of sperm, which reflects mtDNA content, has been observed to exhibit a negative association with fertilization rates^33^. However, it is important to clarify that investigating this particular aspect was not within the scope of our study, and will be explored in future research.

Extensive research has been conducted in the past decade regarding the significance of DFI, with numerous meta-analyses and systematic reviews elucidating the influence of sperm DNA damage on clinical outcomes subsequent to IVF/ICSI^32,34,35^. Nevertheless, it is imperative to underscore that the routine adoption of sperm DNA fragmentation testing for infertility assessment is not endorsed by guidelines, owing to several factors. Primarily, the reliability of the results may be compromised by various factors, including sampling methods, detection techniques, and reagents. Consequently, the uncertainty surrounding the reliability of the results undermines the utility of this testing method. Additionally, the limited availability of high-quality research investigating the association between sperm DNA fragmentation index and fertility outcomes contributes to the ongoing controversy surrounding the clinical significance and value of its detection.

The analysis revealed that the smooth fitting curve provides evidence of a positive correlation between miscarriage rates and DFI, as well as a negative correlation between birth weight and DFI. However, the smooth fitting curve also indicates the absence of a significant nonlinear association between miscarriage rates and DFI (OR 0.902; 95% CI 0.883–0.919; P < 0.001). There appears to be a threshold inflection point between these variables, but further research is required to determine the specific threshold value. Similarly, the study findings demonstrate that there is no linear relationship between birth weight and DFI (OR 1.098; 95% CI 1.083–1.106; *P* < 0.001), but rather that DFI has an impact on birth weight. Based on this study, pregnant couples can be offered more accurate and detailed information about pregnancy outcomes and offspring safety. Nevertheless, certain limitations were identified within the current study, including^36,37^: (1) The retrospective nature of the study introduces the possibility of selection bias, despite efforts made to screen eligible participants and control for confounding variables. Consequently, complete avoidance of this bias remains challenging. (2) The research subjects in this study exclusively consisted of Chinese patients undergoing IVF/ICSI, thereby limiting the generalizability and applicability of the research findings.

In conclusion, sperm DFI affect the miscarriage rates and birth weight in assisted reproductive technology, the smooth fitting curve analysis demonstrates a positive association between DFI and miscarriage rates, as well as a negative association between DFI and birth weight. Nevertheless, this study is limited by its retrospective design, thus necessitating confirmation through multicenter and randomized controlled trials to validate the findings.

## Data Availability

The data presented in this study are available on request from the corresponding author.
